# Aberration correction—impact on image quality and chamber quantification in transthoracic echocardiography

**DOI:** 10.1093/ehjimp/qyae140

**Published:** 2024-12-18

**Authors:** Erik Andreas Rye Berg, Torvald Espeland, Håvard Dalen, Bjørnar Grenne, Tore Grüner Bjåstad, Espen Holte, Svein-Erik Måsøy

**Affiliations:** Department of Circulation and Medical Imaging, Faculty of Medicine and Health Science, Norwegian University of Science and Technology, Prinsesse Kristinas gate 3, Trondheim 7030, Norway; Clinic of Cardiology, St. Olavs hospital, Trondheim University Hospital, Prinsesse Kristinas gate 3, Trondheim 7030, Norway; Department of Circulation and Medical Imaging, Faculty of Medicine and Health Science, Norwegian University of Science and Technology, Prinsesse Kristinas gate 3, Trondheim 7030, Norway; Clinic of Cardiology, St. Olavs hospital, Trondheim University Hospital, Prinsesse Kristinas gate 3, Trondheim 7030, Norway; Department of Circulation and Medical Imaging, Faculty of Medicine and Health Science, Norwegian University of Science and Technology, Prinsesse Kristinas gate 3, Trondheim 7030, Norway; Clinic of Cardiology, St. Olavs hospital, Trondheim University Hospital, Prinsesse Kristinas gate 3, Trondheim 7030, Norway; Department of Circulation and Medical Imaging, Faculty of Medicine and Health Science, Norwegian University of Science and Technology, Prinsesse Kristinas gate 3, Trondheim 7030, Norway; Clinic of Cardiology, St. Olavs hospital, Trondheim University Hospital, Prinsesse Kristinas gate 3, Trondheim 7030, Norway; Department for Research and Development, GE Vingmed Ultrasound, Strandpromenaden 45, Horten 3183, Norway; Department of Circulation and Medical Imaging, Faculty of Medicine and Health Science, Norwegian University of Science and Technology, Prinsesse Kristinas gate 3, Trondheim 7030, Norway; Clinic of Cardiology, St. Olavs hospital, Trondheim University Hospital, Prinsesse Kristinas gate 3, Trondheim 7030, Norway; Department of Circulation and Medical Imaging, Faculty of Medicine and Health Science, Norwegian University of Science and Technology, Prinsesse Kristinas gate 3, Trondheim 7030, Norway; Department for Research and Development, GE Vingmed Ultrasound, Strandpromenaden 45, Horten 3183, Norway

**Keywords:** image quality, reproducibility, aberration correction, transthoracic echocardiography, chamber quantification, image coherence

## Abstract

**Aims:**

To improve image quality (IQ) in echocardiography, an aberration correction (AC) algorithm has recently been implemented in commercial scanners. We aimed to study (i) the correlation of a subjective IQ-score and an objective IQ-metric [global image coherence (GIC)], (ii) if AC improved IQ; (iii) if AC affected average values and interobserver agreement of left ventricular (LV) size, LV longitudinal strain, and left atrial (LA) volume.

**Methods and results:**

From 50 adult patients, where 45 (90%) had cardiovascular disease, unprocessed image data (channel data) were acquired from six standard transthoracic views. The data were processed with and without AC, resulting in 300 pairs of cine-loops. The cine-loops were randomly presented one-by-one to two blinded raters experienced in echocardiography. Both raters scored IQ subjectively from 1 (poor) to 4 (very good) and quantified LV dimensions, volumes and longitudinal strain, and LA volume. IQ-score correlated with GIC, Spearman *rho* 0.72, *P* < 0.001. AC improved median IQ-score from 2.5 to 3.0 (Wilcoxon signed rank: *P* < 0.001). The differences in average values of LV size, LV longitudinal strain, or LA volume with and without AC were not statistically significant and numerically minimal. Measured by intraclass correlation, interobserver agreement of these values was not significantly affected by AC.

**Conclusion:**

Image quality-score strongly correlated with GIC. Aberration correction improved IQ. However, AC did not lead to statistically significant changes in average values or interobserver agreement of LV size, LV longitudinal strain or LA volume quantification. Likely, the major benefit of AC is enhanced visualization of anatomical details.

## Graphical Abstract

**Graphical abstract:**
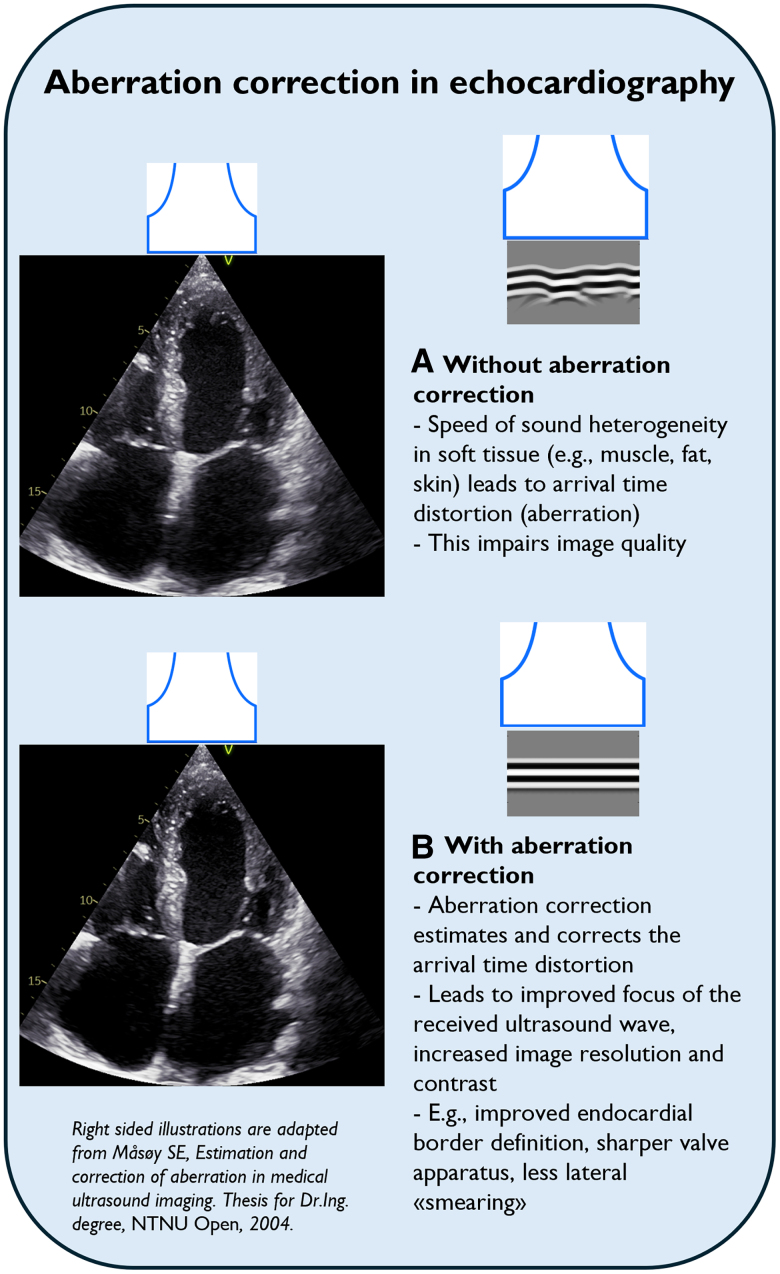

## Introduction

The speed of sound is a fundamental parameter in echocardiography. The assumed speed of sound in echocardiography is 1540 m/s, which represents an average value across soft tissues.^1^ However, the speed of sound differs between different soft tissues.^1–4^ Importantly, the erroneous assumption of constant speed of sound degrades image quality (IQ).^5,6^

The first step of image construction in modern ultrasound linear or matrix array transducers is focusing. Due to differences in the propagation distance, the ultrasound wave arrives at the different transducer elements at different time points. By correcting the arrival time differences, the wave can be summed over all elements to provide a strong signal from a given point. If the speed of sound is constant, such focusing leads to higher resolution and better contrast. However, heterogeneity for the speed of sound across different soft tissues leads to arrival time distortion, which is often termed arrival time error or aberration. Unless corrected, this results in a lower coherence in the summation process of the transducer elements, leading to impaired IQ by decreasing both resolution and contrast.^7^

**Figure 1:**
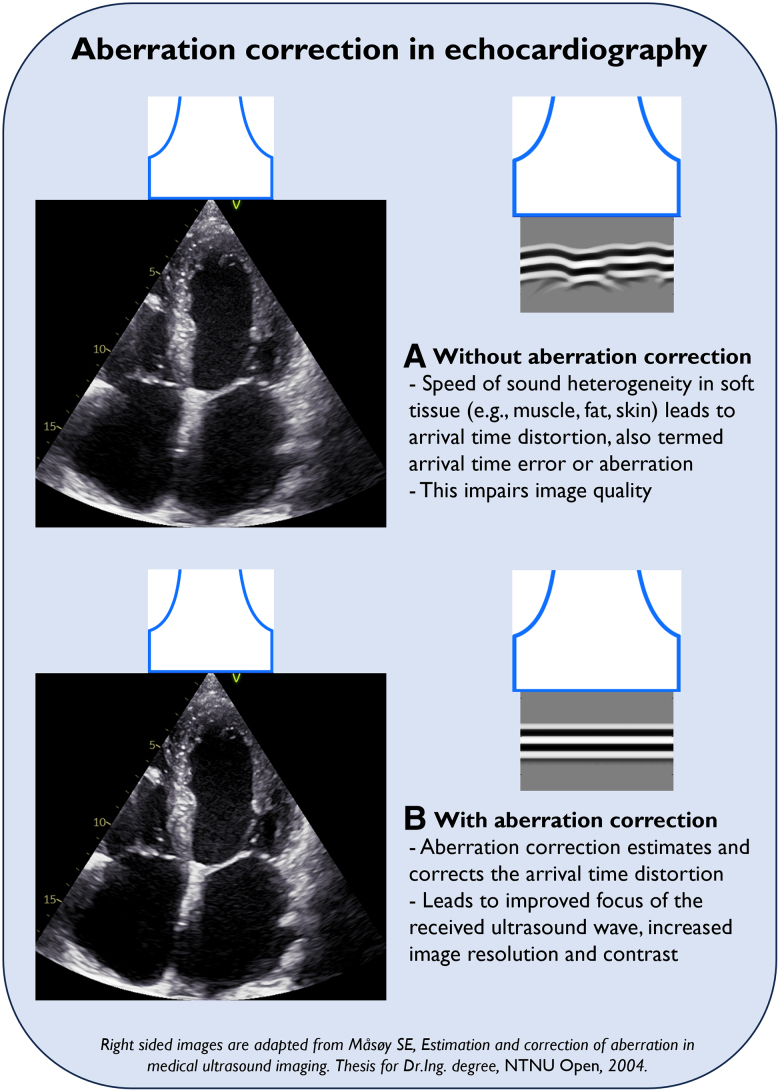
Aberration correction in echocardiography. The figure displays an apical 4-chamber view image from the same recording with conventional processing (*A*) and with AC (*B*). In this example, AC improved the definition of the endocardial border, perhaps most pronounced in the left atrium. Moreover, anatomical details of the mitral valve and the subvalvular apparatus appear sharper with less lateral ‘smearing’.

Optimizing IQ in echocardiography is essential for adequate diagnostic accuracy both in clinical care and research.^8^ To improve IQ, an aberration correction (AC) algorithm has recently been developed and has been made available on clinical scanners.^9^ The algorithm corrects the arrival time distortion caused by heterogenous tissue speed of sound, resulting in improved image coherence (*Figure 1*). The algorithm has previously been demonstrated to improve IQ in echocardiography measured by a global image coherence (GIC) metric.^10^ Global image coherence is a quantitative metric for *in vivo* IQ that has been shown to correlate well with conventional IQ-metrics.^10^ Furthermore, from blinded side-by-side evaluation, experts preferred AC over non-corrected cine-loops.^9^

In this study, the aims were the following:

1. To study the correlation between a subjective IQ-score and GIC
2. To study if AC improved IQ-score when evaluated in a blinded random order without side-by-side comparison
3. To investigate the effect of AC on the average values and interobserver agreement of quantification of left ventricular (LV) dimensions, volumes and strain, and left atrial (LA) volume

## Methods

### Study population

In this prospective study, we recruited 50 patients at the European Association of Cardiovascular Imaging-accredited echocardiography laboratory at the Clinic of Cardiology, St. Olavs hospital, Trondheim, Norway. Participants were either hospitalized patients or patients meeting for echocardiography at the outpatient clinic. All had a clinical indication for transthoracic echocardiography, were >18 years old, and provided written informed consent. Patients who were unable to provide informed consent or hemodynamically unstable were not invited to participate. The patients were included consecutively during three working days in May 2022.

### Data acquisition

Echocardiographic data were recorded by two experienced physician echocardiographers (TE and EARB) using a Vivid E95 clinical scanner and a 4Vc-D probe (GE Vingmed Ultrasound AS, Horten, Norway). The scanner software was modified to store unprocessed image data (channel data). With a frame rate of 40 frames per second, data were stored prospectively. A total number of 64 frames (128 frames in cases with bradycardia) were stored and later analysed offline. For all included cases, separate acquisitions were performed for six standard views: the parasternal long-axis view, LV-focused apical 4-chamber, 2-chamber, and long-axis views, as well as LA-focused apical 4-chamber and 2-chamber views.

### AC algorithm

The AC algorithm estimates and corrects arrival time distortion (aberration) in 2D echocardiography.^9^ The algorithm is applied using the 4Vc-D matrix array probe, which contains a sub-aperture processor (SAP) structure, where element signals are pre-beamformed within the probe into 192 channels. The Vivid E95 system can store and separate these data (referred to as channel data) from each SAP, prior to both beamforming and image processing. The purpose of this process is to estimate and correct arrival time distortion across the 192 SAP channels. By applying the estimated arrival time distortion in the beamforming process, an increase in signal intensity and improved resolution and contrast in the final presented image loop is achieved independently of frame rate, heart rhythm, and heart rate. Comprehensive details have recently been published.^9^

### Global image coherence

The method for GIC estimation has been presented in detail previously.^10^ In brief, from channel data, GIC was defined as the spatial average coherence value from all data pixels forming the image. The reported GIC value was the temporal average of GIC from all frames in a cine-loop. A higher GIC represents a higher image coherence and generally a higher IQ. However, there are no established reference values for GIC. We report GIC per view and global GIC as the average from all six recorded views.

### Data post-processing

The acquired data were post-processed as previously described, using a version of the Vivid E95 cSound beamforming and image processing software implemented in MATLAB (The Mathworks, Inc., Natick, MA, USA).^9^ This process yielded two types of cine-loops from each recording:

- Cine-loops with conventional image processing without AC (NoAC).
- Cine-loops with AC, with adequate gain compensation.

After deidentification, all cine-loops were presented in a random order for analyses.

### Echocardiographic analysis

All echocardiography data were analysed by two experienced observers (T.E. and E.A.R.B.) using EchoPac v. 204 (GE HealthCare, Horten, Norway) 12 months after inclusion. A total of 600 cine-loops were analysed (300 AC and 300 NoAC).

**Figure 2:**
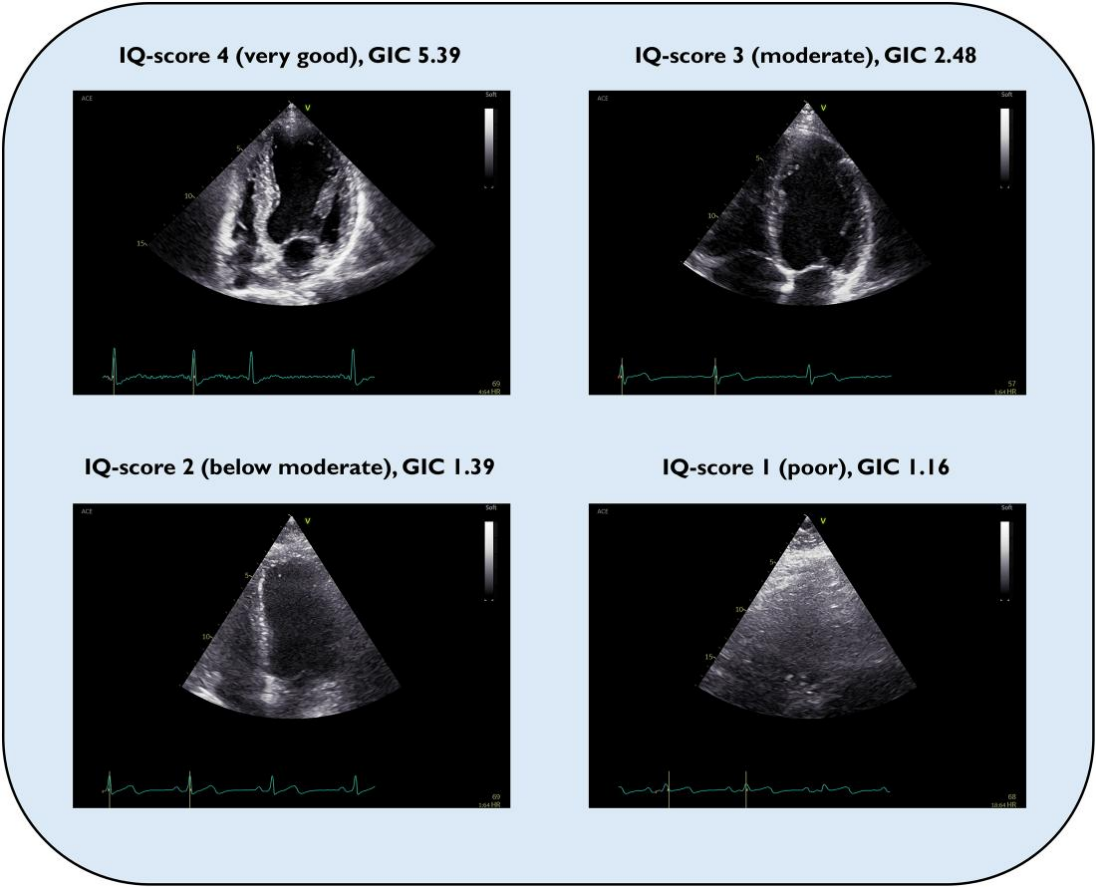
Image quality score and GIC. Image quality-score was rated subjectively from 1 to 4 (4 best) from each cine-loop by two observers. The examples above shows end-diastolic images from LV-focused apical 4-chamber view where both observers agreed on the IQ-score. GIC is an objective measure on the global coherence in the ultrasound image. A low GIC-value is associated with high degree of aberration. The reported GIC is the average value from the complete recording in each view.

Image quality was evaluated subjectively for each cine-loop, primarily focusing on global endocardial border visualization. The two readers scored IQ independently from 1 to 4 (1, poor; 2, below moderate, 3; moderate and 4; very good). *Figure 2* displays examples of the different IQ-score categories. The IQ-scores presented throughout the manuscript are averages from both observers’ individual scores. The IQ-score is given per view unless specified as a global IQ-score, the latter being the average from all six views.

The timing of end-diastole and end-systole was pre-assigned by one observer (E.A.R.B.). During analyses, each image loop was presented individually, and the link between the different cine-loops from each patient was not available to the blinded raters. This means that strain and volumes were assessed only in single view and not from biplane or triplane assessment. From the parasternal long-axis view, septal and posterior wall thickness and internal diameter of the LV were measured manually at end-diastole. Furthermore, from manual tracing of the endocardial border LV-focused apical 4- and 2-chamber views, end-diastolic volume (EDV) and end-systolic volume (ESV) were estimated. Single-view LV longitudinal strain was estimated from LV-focused apical 4-, 2-chamber and long-axis views using the semi-automatic 2DS function. Left atrium ESV was estimated from LA-focused 4- and 2-chamber views. All analyses were performed in accordance with current recommendations for cardiac chamber quantification in adults,^11^ albeit with a liberal approach regarding feasibility.

### Statistical analyses

Dichotomous data are presented as numbers (percentages). Depending on the distribution, continuous variables are presented as mean ± SD or median (25–75%) unless specified otherwise. Normal distribution was evaluated by QQ-plots, histograms and formally using the Shapiro–Wilk test. Comparisons between groups were analysed by Wilcoxon Signed Ranks and Spearman rank correlation tests as appropriate. Interobserver agreement was investigated by Bland–Altman statistics and a two-way mixed intraclass correlation model for absolute agreement, reported as bias [95% limits of agreement (LoA)] and intraclass correlation coefficient (ICC), respectively. Fischer’s *z*-transformation was applied to compare correlation coefficients. A two-sided value of *P* < 0.05 was considered statistically significant. Data were processed and analysed in SPSS (version 29.0, IBM, New York, NY, USA).

## Results

### Patient characteristics

Patient characteristics are provided in *Table 1.* The median age was 65 years, 17 (34%) were females, and the median body mass index was 27 kg/m^2^. Cardiovascular disease was present in 45 (90%) of the participants. Nine (18%) patients had atrial fibrillation during the recordings. There was a wide range for the measures of LV size and strain and LA volume (*Tables 2–4*), e.g. 4-chamber LV EDV ranged 47–352 mL.

**Table 1:** Patient characteristics at the time of inclusion

Age (years)	65 (55–79)
Females	17 (34%)
Height (cm)	176 ± 9
Weight (kg)	85 ± 15
Body mass index (kg/m^2^)	26.8 (24.3–29.9)
Body surface area (m^2^)	2.01 ± 0.19
Primary cardiovascular condition	
No cardiovascular disease	5 (10%)
Non-ischemic cardiomyopathy	4 (8%)
Coronary artery disease	17 (34%)
Valvular heart disease	10 (20%)
Arrhythmia	6 (12%)
Other cardiovascular disease^a^	8 (16%)
Total	50 (100%)

^a^Hypertension, infectious endocarditis, pericardial disease, persistent foramen ovale, pulmonary hypertension, and thoracic aortic aneurysm.

**Table 2:** LV end-diastolic dimensions with and without AC

		NoAC		AC	
		Observer 1	Observer 2	Observer 1	Observer 2
**IVSd (mm), *n* = 49**	Mean ± SD	11 ± 2	11 ± 2	11 ± 3	11 ± 2
	Median (25%–75%)	11 (9–13)	11 (10–13)	11 (10–13)	11 (9–13)
	Min–max	8–16	7–18	7–19	7–18
	Bias (95% LoA)	0 (−3, 3)		0 (−2, 3)	
	ICC, absolute agreement	0.85		0.88	
		*P* = 0.57			

**LVIDd (mm), *n* = 49**	Mean ± SD	52 ± 10	53 ± 9	52 ± 9	53 ± 9
	Median (25%–75%)	51 (46–57)	52 (47–58)	51 (45–57)	51 (47–59)
	Min–max	36–78	34–77	35–78	36–80
	Bias (95% LoA)	−1 (−7, 5)		−1 (−5, 3)	
	ICC, absolute agreement	0.94		0.97	
		*P* = 0.14			

**LVPWd (mm), *n* = 49**	Mean ± SD	9 ± 1	8 ± 2	9 ± 2	9 ± 2
	Median (25%–75%)	9 (8–9)	8 (7–9)	9 (8–10)	9 (8–9)
	Min–max	6–13	6–13	6–13	6–13
	Bias (95% LoA)	0 (−3, 4)		0 (−2, 3)	
	ICC, absolute agreement	0.32		0.53	
		*P* = 0.21			

IVSd, end-diastolic interventricular septal thickness; LVIDd, end-diastolic left ventricular internal diameter; LVPWd, end-diastolic left ventricular posterior wall thickness.

**Table 3:** LA and LV volume quantification with and without AC

		LV-focused apical 2-chamber view (*n* = 49)				LV-focused apical 4-chamber view (*n* = 49)			
		NoAC		AC		NoAC		AC	
		Observer 1	Observer 2	Observer 1	Observer 2	Observer 1	Observer 2	Observer 1	Observer 2
**LV EDV (mL)**	Mean ± SD	140 ± 70	150 ± 69	146 ± 76	149 ± 66	140 ± 74	142 ± 70	141 ± 73	147 ± 71
	Median (25–75%)	127 (95–160)	134 (96–177)	127 (86–184)	135 (98–188)	120 (85–156)	124 (99–165)	123 (89–170)	126 (98–172)
	Min–max	48–398	61–383	41–395	56–348	45–340	48–363	45–348	59–396
	Bias (95% LoA)	−9 (−60, 42)		−3 (−50, 43)		−3 (−42, 37)		−6 (−47, 35)	
	ICC, absolute agreement	0.93		0.95		0.96		0.96	
		*P* = 0.44				*P* = 0.82			

**LV ESV (mL)**	Mean ± SD	78 ± 68	83 ± 68	81 ± 72	80 ± 64	78 ± 69	79 ± 68	77 ± 67	81 ± 68
	Median (25–75%)	60 (37–77)	57 (39–89)	55 (32–96)	56 (38–91)	49 (34–76)	54 (39–83)	52 (34–83)	55 (43–89)
	Min–max	15–344	10–337	15–347	16–318	17–291	18–309	16–286	19–320
	Bias (95% LoA)	−5 (−46, 36)		0 (−32, 33)		−1 (−42, 40)		−3 (−42, 35)	
	ICC, absolute agreement	0.95		0.97		0.95		0.96	
		*P* = 0.22				*P* = 0.87			

		LA-focused apical 2-chamber view (*n* = 50)				LA-focused apical 4-chamber view (*n* = 50)			
		NoAC		AC		NoAC		AC	
		Observer 1	Observer 2	Observer 1	Observer 2	Observer 1	Observer 2	Observer 1	Observer 2
**LA ESV (mL)**	Mean ± SD	76 ± 37	75 ± 41	76 ± 41	76 ± 43	80 ± 45	74 ± 38	78 ± 43	75 ± 41
	Median (25–75%)	64 (50–95)	61 (46–96)	61 (47–95)	65 (49–93)	67 (50–98)	69 (47–95)	64 (49–95)	63 (45–99)
	Min–max	32–207	27–230	30–224	25–246	23–280	26–230	26–266	22–242
	Bias (95% LoA)	2 (−18, 21)		0 (−21, 21)		6 (−18, 31)		3 (−20, 27)	
	ICC, absolute agreement	0.97		0.97		0.95		0.96	
		*P* = 1.00				*P* = 0.50			

**Table 4:** LV longitudinal strain quantification with and without AC

		LV longitudinal strain (%)			
		NoAC		AC	
		Observer 1	Observer 2	Observer 1	Observer 2
**Apical 4-chamber view** **(*n* = 44)**	Mean ± SD	−16 ± 6	−15 ± 5	−16 ± 6	−15 ± 6
	Median (25–75%)	−17 (−20 to −12)	−16 (−18 to −11)	−17 (−20 to −12)	−16 (−19 to −12)
	Min–max^a^	−25 to 0	−23 to 0	−28 to 0	−26 to 0
	Bias (95% LoA)	−1 (−4, 3)		−1 (−4, 3)	
	ICC, absolute agreement	0.95		0.96	
		*P* = 0.70			
**Apical 2-chamber view** **(*n* = 37)**	Mean ± SD	−14 ± 5	−14 ± 5	−14 ± 6	−14 ± 6
	Median (25%–75%)	−16 (−18 to −10)	−15 (−18 to −11)	−17 (−19 to −10)	−16 (−19 to −10)
	Min–max^a^	−23 to −4	−22 to 0	−22 to 0	−24 to 0
	Bias (95% LoA)	−0 (−4, 3)		−0 (−3, 3)	
	ICC, absolute agreement	0.94		0.96	
		*P* = 0.41			
**Apical long-axis view** **(*n* = 33)**	Mean ± SD	−13 ± 6	−13 ± 5	−13 ± 6	−13 ± 6
	Median (25%–75%)	−15 (−17 to −7)	−15 (−17 to −9)	−16 (−17 to −8)	−14 (−18 to −8)
	Min–max^a^	−22 to 0	−22 to 0	−26 to 0	−23 to 0
	Bias (95% LoA)	0 (−3, 4)		0 (−5, 5)	
	ICC, absolute agreement	0.96		0.92	
		*P* = 0.23			

^a^Longitudinal strain between −3.5% and 0% was assigned value 0% by the software.

### Feasibility

Image quality-scoring was feasible in all recordings of both NoAC and AC cine-loops. Quantification of LV end-diastolic wall thickness, internal diameter, EDV, and ESV was feasible in 49 (98%) subjects. LV longitudinal strain was feasible in 44 (88%) subjects in 4-chamber view, 37 (74%) in 2-chamber view, and 33 (66%) in apical long-axis view. The main reason for the non-feasibility of strain analysis was incomplete cardiac cycles, i.e. not including end-diastole to end-diastole. Left atrium volume quantification was feasible in all subjects.

### Image quality

**Figure 3:**
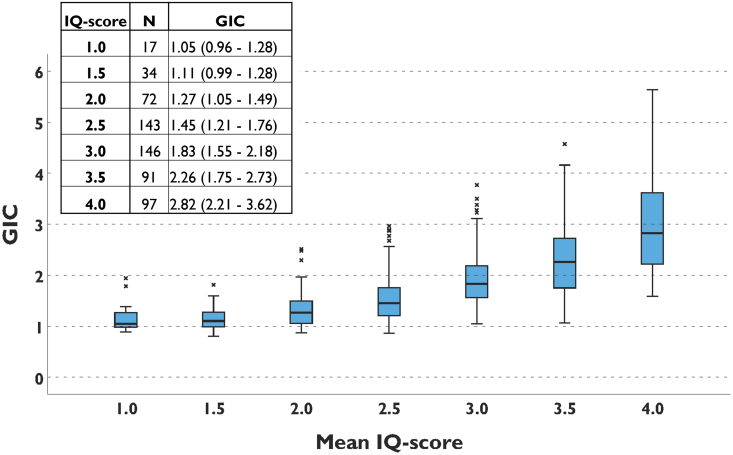
Boxplot of IQ score in relation to GIC. All data from both conventional processing and AC are included in the figure. There is a positive correlation between two-rater average IQ-score and GIC, Spearman *rho* 0.72, *P* < 0.001. The number of images (N) and median (25%–75%) GIC for each IQ-score category are presented in the top left corner.

There was a strong positive correlation between the subjective IQ-score and GIC, *rho* = 0.72, *P* < 0.001 (*Figure 3*).

**Figure 4:**
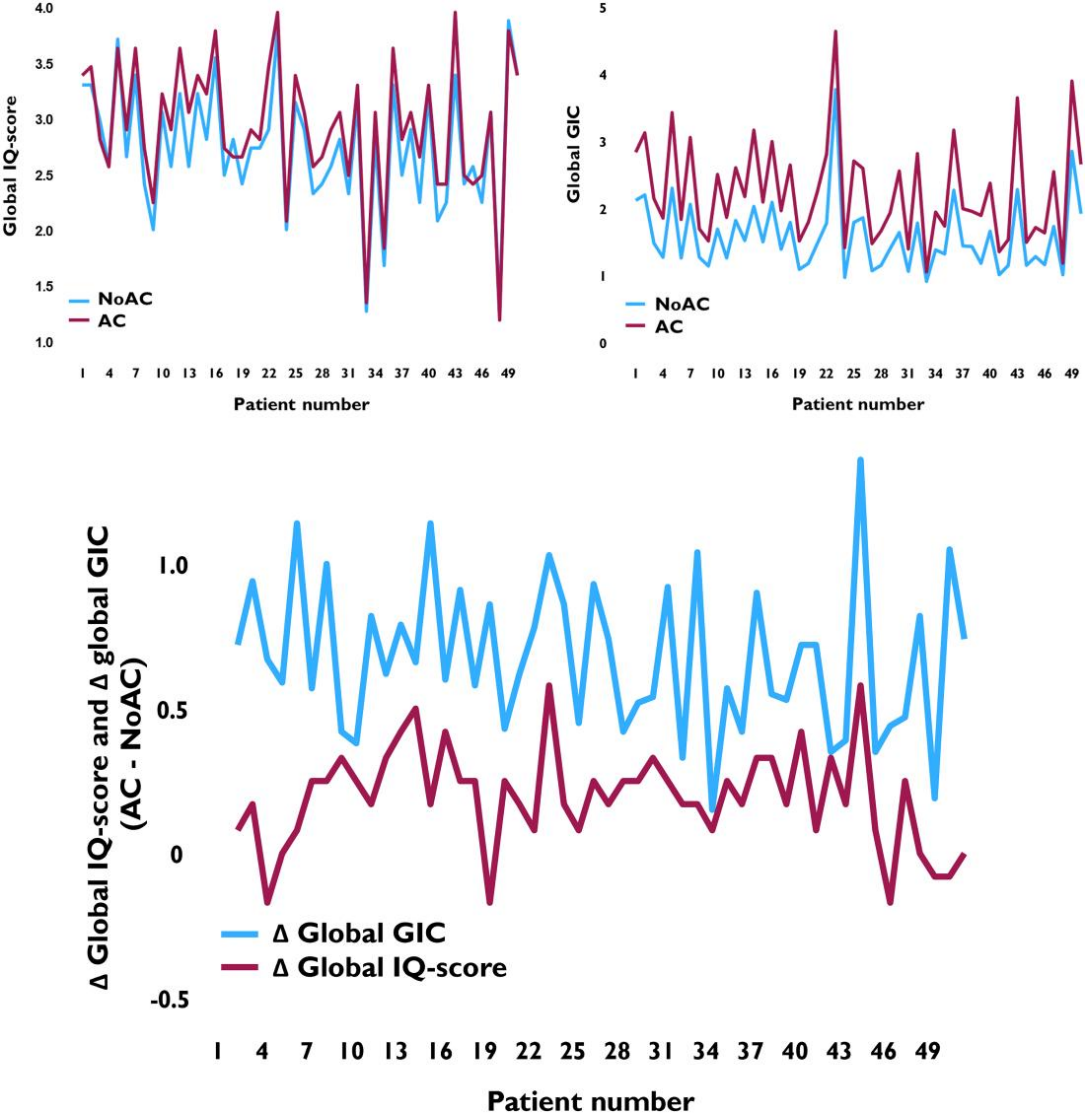
Individual global IQ score and GIC for all 50 patients. Note that Δ global IQ-score and Δ global GIC >0 both indicate improved IQ with AC compared with conventional image processing (NoAC).

*Table 5* displays the IQ-scores for NoAC and AC cine-loops for all views. Median IQ-score was 3.0 in AC recordings and 2.5 in NoAC recordings. Furthermore, in 41 (82%) subjects, AC provided the better overall IQ-score (*P* < 0.001) (*Figure 4*). This finding was consistent across all views.

**Table 5:** IQ-score for different views with and without AC

		*n*	Mean		Median		Wilcoxon signed ranks test			
			NoAC	AC	NoAC	AC	AC best	NoAC best	Tie	*P*
**Parasternal long-axis view**		50	3.0	3.2	3.0	3.5	23	4	23	<0.001
**LA apical views**	**2-chamber**	50	2.7	3.0	2.5	3.0	24	6	20	<0.001
	**4-chamber**	50	3.0	3.1	3.0	3.0	17	4	29	0.014
**LV apical views**	**2-chamber**	50	2.6	2.7	2.5	2.5	20	8	22	0.034
	**4-chamber**	50	2.8	3.0	3.0	3.0	20	4	26	<0.001
	**Long-axis**	50	2.5	2.7	2.5	3.0	20	7	23	0.004
**Total**		300	2.8	3.0	2.5	3.0	124	33	143	<0.001
**Global (6 views)**		50	2.8	3.0	2.8	2.9	41	6	3	<0.001

*Figure 4* displays individual global IQ-score and global GIC for all patients. The individual global IQ-score corresponded for NoAC and AC and ranged from 1.2 to 4.0. A similar covariation was seen for global GIC. Aberration correction improved global GIC in all individuals.

### Chamber quantification

Average measurements for LV wall thickness, internal diameter, volumes, and strain are reported in *Tables 2–4*. Similarly, average measurements of LA volumes are reported in *Table 3*. The differences in average values from AC and NoAC were minimal and not statistically significant.

Details of the interobserver agreement for quantitative measures of LV wall thickness, internal diameter, volumes, and strain, and LA volumes are also included in *Tables 2–4*. Bland–Altman plots for the interobserver agreement are presented in Supplementary data online, *F*igures *S1*–*S5*. When comparing interobserver agreement with and without AC, the ICC was slightly higher for AC recordings in 10 of the 14 comparisons (2 ties). However, none of the differences were statistically significant. Moreover, the same trend was observed for the width of the 95% LoA, which was narrower in AC recordings in 9 of the 14 comparisons (1 tie).

## Discussion

In this study, we have investigated the correlation between IQ-score and GIC, and the effect of AC on IQ and interobserver agreement for important echocardiographic measures of the LA and LV. We found that IQ-score and GIC were strongly correlated. Moreover, AC improved IQ compared with NoAC. However, AC did not improve interobserver agreement compared with NoAC. Finally, there were no systematic differences between AC and NoAC with respect to average values for the quantitative measures from the LA and the LV.

### Study population and statistics

The study population was recruited from a cardiology ward and outpatient clinic, without selection based on diagnosis or IQ. This led to a clinically relevant study population with wide ranges in chamber sizes and function. Moreover, as in an unselected clinical population, IQ ranged from excellent to very poor.

As expected in a diseased population, several parameters were not normally distributed. Moreover, heteroscedasticity was evident for the interobserver assessment of LA and LV volumes. Nevertheless, for the assessment of interobserver agreement, we have opted for parametric analyses without any data transformation, as a main purpose of the study was comparison of interobserver agreement with and without AC. Importantly, this means that the specific numerical values for LoA and ICC reported in this study do not give a true reflection of the data and should not be used for other purposes.

### Feasibility

In general, we applied a liberal approach to performing analyses even in poor IQ, and only a few cine-loops were excluded from chamber quantification due to poor IQ. Consequently, the feasibility was high for dimensions and volumetric indices. However, the feasibility for strain analyses was markedly lower as the recordings were limited by the number of frames and did not necessarily include a complete cardiac cycle (*Videos 1*, *6*) The data were collected from a prospective, non-ECG gated recording setting combined with limitations in the number of frames stored due to the large size of the channel data. As one single image loop of 64 frames (40 frames per second) approximated 3 gigabytes of storage space, we had to restrict the total number of frames recorded related to the scanner’s internal memory and storing capacities.

### Image quality

In a population with highly heterogeneous IQ, we have demonstrated a strong positive correlation between the subjective IQ-score and GIC. This validates the use of GIC as a measure of IQ and supports the use of either of these parameters for IQ evaluation in echocardiography.

The subjective scoring of 600 randomly ordered cine-loops by each of the two blinded raters showed better IQ-score with AC compared with NoAC. This finding was highly significant and consistent across all views. This adds to the findings from the previous side-by-side comparison study, where AC recordings were preferred over NoAC.^6^ The effect of AC on IQ-score was heterogeneous both within and between subjects. However, the variation in IQ between subjects was substantially larger than the effect of AC within each subject.

### Chamber quantification

**Figure 5:**
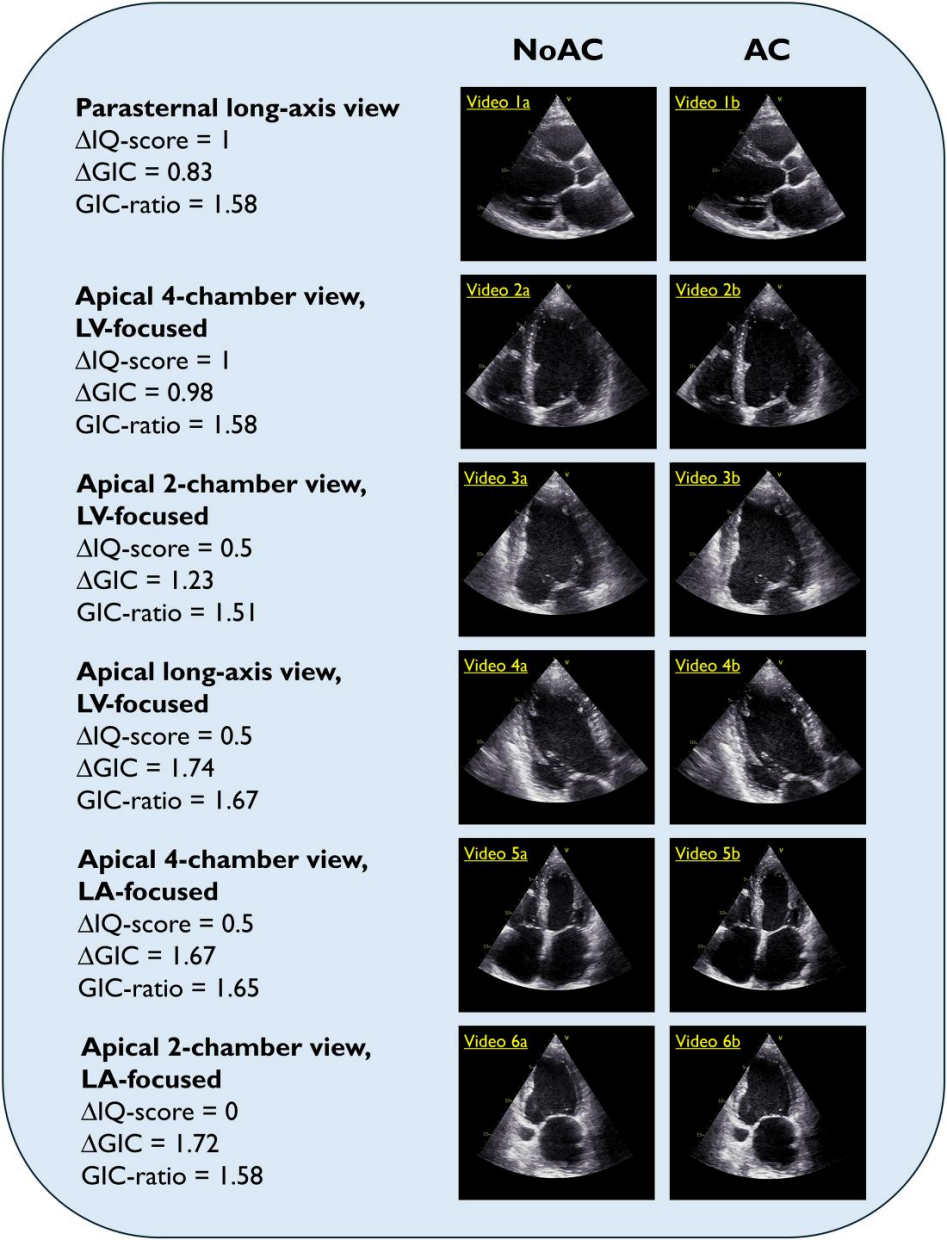
Effect of AC. These examples are all from the same case, displaying images/videos (speed 50%) from six views with both conventional processing (NoAC) and AC. The corresponding changes in IQ score and GIC are shown on the left side. ΔIQ-score >0, ΔGIC >0 and GIC-ratio >1 indicates improved IQ from AC. In these examples, AC improves the contrast and visualization of the endocardial border and the mitral valve apparatus with less lateral ‘smearing’. *Videos 1* and *6* are examples of recordings of incomplete cardiac cycles. LA, left atrium; LV: left ventricle.

Interobserver variability in chamber quantification is substantial.^12–16^ AC led to improvement in IQ, which may include a more defined endocardial delineation (*Figures 1* and *5, Videos 1–6*). The results in this study showed a weak trend toward better interobserver agreement after AC; however, this was not statistically significant. A larger sample size might reveal statistically significant differences. Moreover, experience-based temporal and spatial interpolation to compensate for missing data may attenuate the effect of AC on chamber quantification. Overall, it is likely that the effect from AC on interobserver variability in chamber quantification is minor, at least among experienced raters.

Moreover, in this study, AC had a negligible effect on the average values for quantitative measures of LV size and strain and LA volume, limiting the benefits of a more rigid comparison against another methodology such as cardiac magnetic resonance imaging.

### Future perspectives

The most pronounced effect of AC was improved contrast and sharpness of anatomical details, including better visualization of the endocardial border. This is especially evident at larger depths, e.g. better delineation of the mitral valvular apparatus as shown in *Figures 1* and *5, Videos 1–6*. However, AC did not significantly improve interobserver agreement for manual assessment of chamber size and function in this study. Nevertheless, a larger study with either manual or automated assessment of chamber size and function may provide valuable additional insights.

Due to the enhanced contrast and sharpness of anatomical details, AC has the potential to improve characterization and discrimination of structural pathologies such as tumours, thrombi, vegetations, fistula, perforations, abscesses, and prolapses. Such improvements could in some cases improve the diagnostic yield from transthoracic echocardiography, possibly lowering the demand for transoesophageal echocardiography.

### Limitations

The most important limitation of this study is the small sample size of 50 participants, which may be too small to reveal subtle differences in interobserver agreement in chamber quantification following AC. However, based on the small effect size in the presented results, any such differences are likely to be minor and of limited clinical relevance. Moreover, the study was neither designed nor powered to investigate if specific cardiovascular diseases impact the effect of AC on IQ or interobserver agreement.

The reported values of ICC and 95% LoA in this study are not generalizable, as assumptions including normality and/or homoscedasticity were violated. However, the direct comparisons within this study are still valid.

Feasibility of LV longitudinal strain was low due to the settings used for channel data recordings, effectively reducing the sample size. This could have been improved by implementing ECG-gated timing of recordings or by increasing the number of recorded frames. Following the same issue (*Videos 6*), and a frequently obtained frame rate below the lower limit for LA automated function imaging (GE Vingmed Ultrasound), we have not performed LA strain analyses as part of this study.

The IQ-score was based on a subjective evaluation of the individual cine-loops. Endocardial border detection was the main factor in this generalized score. Application of a more detailed, segmental, or signal-based scoring system may affect the differences seen in IQ-score between AC and NoAC.

## Conclusion

The strong correlation between subjective IQ-score and GIC supports the use of either of these parameters for IQ evaluation in echocardiography. AC of echocardiographic recordings significantly improved IQ compared with NoAC when evaluated by two blinded experienced raters. However, this improvement did not translate to improved interobserver agreement in quantification of LA and LV size and function. Moreover, AC did not significantly affect the average values of these metrics. The main improvement from AC was enhanced visualization of anatomical details. Potentially, the major benefit of AC may not be related to chamber quantification but rather improved imaging of detailed structural pathology.

## Supplementary data

Supplementary data are available at *European Heart Journal - Imaging Methods and Practice* online.

**Conflict of interest:** E.A.R.B. has received a PhD grant and S.E.M. has been employed at Centre for Innovative Ultrasound Solutions (CIUS), where GE Vingmed Ultrasound was an industrial partner. H.D. and B.G. are associated with Precision Health Center for Optimized Cardiac Care (ProCardio), where GE Vingmed Ultrasound is an industrial partner. T.G.B. and S.E.M. hold positions at GE Vingmed Ultrasound. GE Vingmed Ultrasound has provided an Vivid E95-scanner for restriction-free research at our department.

## Consent

The study was approved by the regional committee for medical and health research ethics (REK 2022/282408). All participants received oral and written information on the study and have provided written consent.

## Supplementary Material

qyae140_Supplementary_Data

## Data Availability

The data underlying this study cannot be shared publicly due to limitations in study approval and patient consent.

## Lead author biography

Erik Andreas Rye Berg (b. 1981), MD, graduated from Norwegian University of Science and Technology (NTNU) in 2008. He has performed research at CIUS and NTNU since 2016. Since 2017 he has been a consultant in internal medicine at St. Olavs hospital, Orkdal, Norway. He currently holds a position as fellow in cardiology at St. Olavs hospital, Trondheim, Norway. His main scientific interests are related to myocardial function and valvular heart disease.

Torvald Espeland (b. 1981), MD, graduated from NTNU in 2008. He is currently pursuing the PhD degree with the Department of Circulation and Medical Imaging. He currently holds a position as a fellow in cardiology at the Clinic of Cardiology, St. Olavs Hospital, Trondheim University Hospital, Trondheim. His main interests are cardiac imaging, advanced echocardiography, and cardiovascular physiology and hemodynamics.
