# Supplementary material for: Aberration correction—impact on image quality and chamber quantification in transthoracic echocardiography
Source: Eur Heart J Imaging Methods Pract. 2024 Dec 18;3(1):qyae140. doi: 10.1093/ehjimp/qyae140 (PMC11852281; doi:10.1093/ehjimp/qyae140)
Supplement: qyae140_Supplementary_Data [file qyae140_Supplementary_Data.zip › Supplementary figure legends.docx]

## Supplementary figure legends

***Supplementary figure 1. Bland-Altman plots of interobserver agreement of left ventricular dimensions with conventional image processing (NoAC) and aberration correction (AC).****The dashed lines represent the upper and lower 95% limits of agreement. The solid line represents the bias.*
*IVSd: end-diastolic interventricular septal thickness; LVIDd: end-diastolic left ventricular internal diameter; LVPWd: end-diastolic left ventricular posterior wall thickness*

***Supplementary figure 2. Bland-Altman plots of interobserver agreement of left ventricular (LV) 2-chamber volumes with conventional image processing (NoAC) and aberration correction (AC).****The dashed lines represent the upper and lower 95% limits of agreement. The solid line represents the bias.
EDV: end-diastolic volume; ESV: end-systolic volume*

***Supplementary figure 3. Bland-Altman plots of interobserver agreement of left ventricular (LV) 4-chamber volumes with conventional image processing (NoAC) and aberration correction (AC).****The dashed lines represent the upper and lower 95% limits of agreement. The solid line represents the bias.
EDV: end-diastolic volume; ESV: end-systolic volume*

***Supplementary figure 4. Bland-Altman plots of interobserver agreement of left atrial (LA) volumes with conventional image processing (NoAC) and aberration correction (AC).****The dashed lines represent the upper and lower 95% limits of agreement. The solid line represents the bias.
A2C: apical 2-chamber view; A4C: apical 4-chamber view; ESV: end-systolic volume*

***Supplementary figure 5. Bland-Altman plots of interobserver agreement of left ventricular (LV) longitudinal strain with conventional image processing (NoAC) and aberration correction (AC).****The dashed lines represent the upper and lower 95% limits of agreement. The solid line represents the bias.
A2C: apical 2-chamber view; A4C: apical 4-chamber view; ALAX: apical long-axis view*
