# Supplementary figures and images for: Aberration correction—impact on image quality and chamber quantification in transthoracic echocardiography
Source: Eur Heart J Imaging Methods Pract. 2024 Dec 18;3(1):qyae140. doi: 10.1093/ehjimp/qyae140 (PMC11852281; doi:10.1093/ehjimp/qyae140)

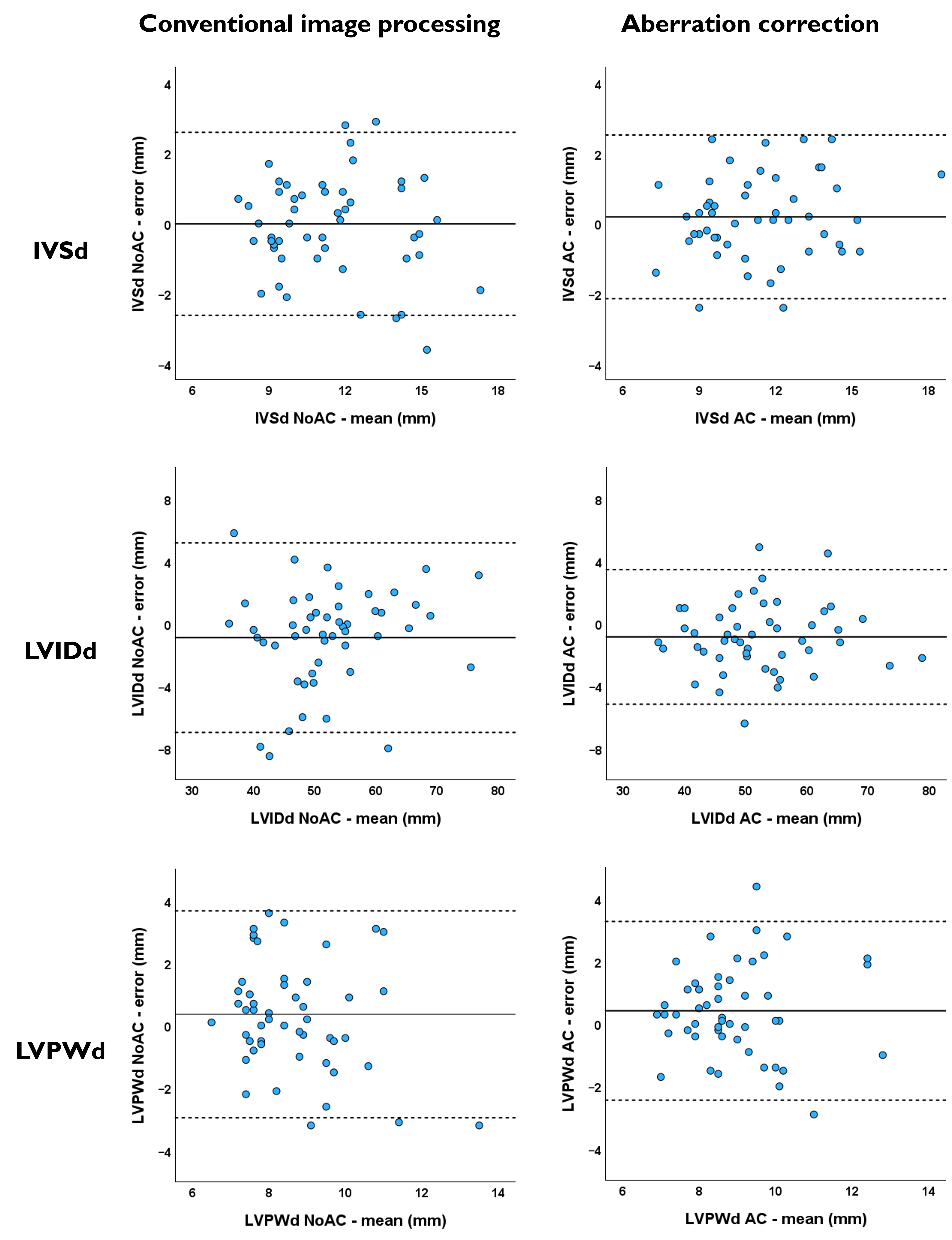

Supplement: qyae140_Supplementary_Data [file qyae140_Supplementary_Data.zip › Supplementary figure 1.tif]

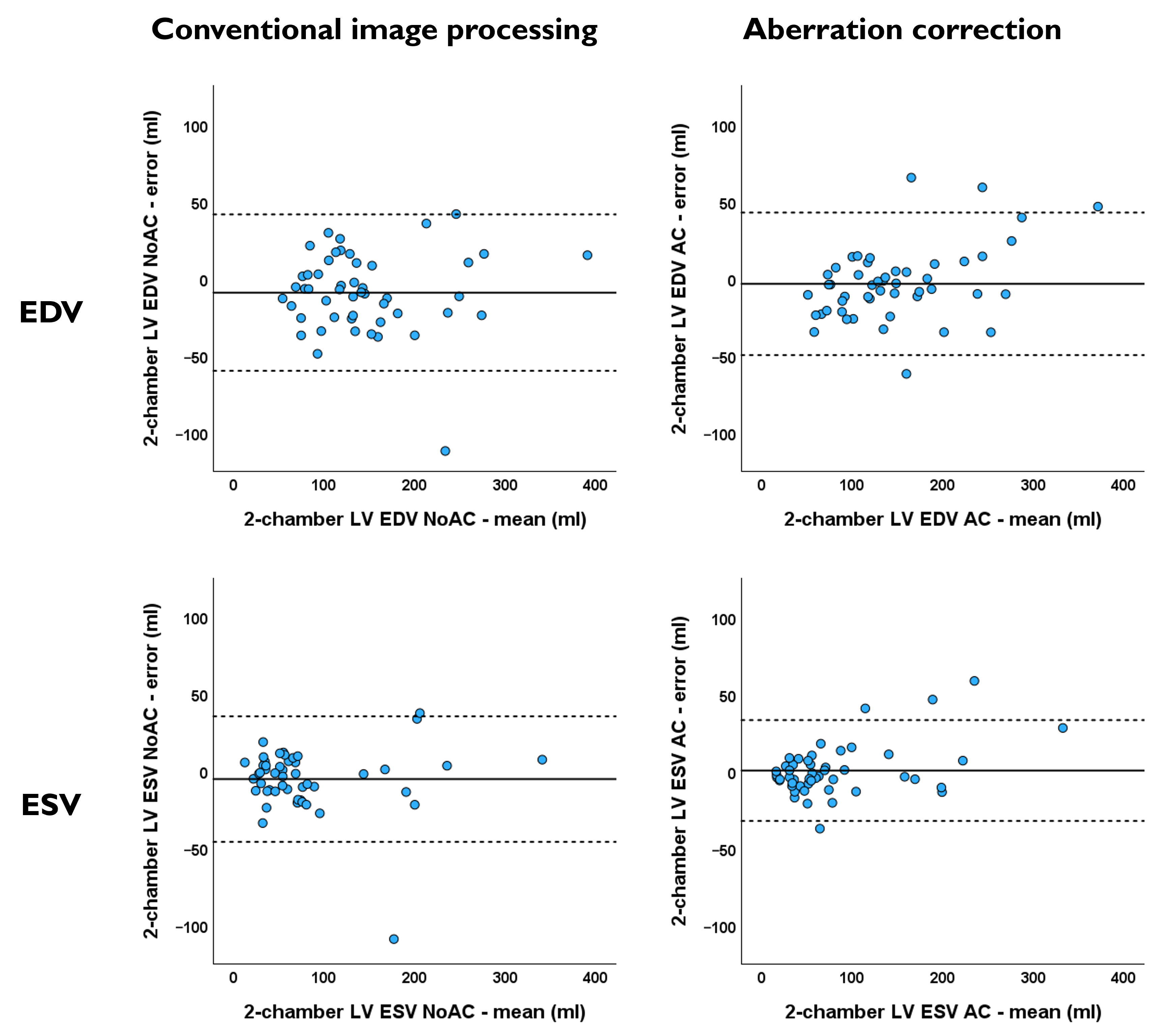

Supplement: qyae140_Supplementary_Data [file qyae140_Supplementary_Data.zip › Supplementary figure 2.tif]

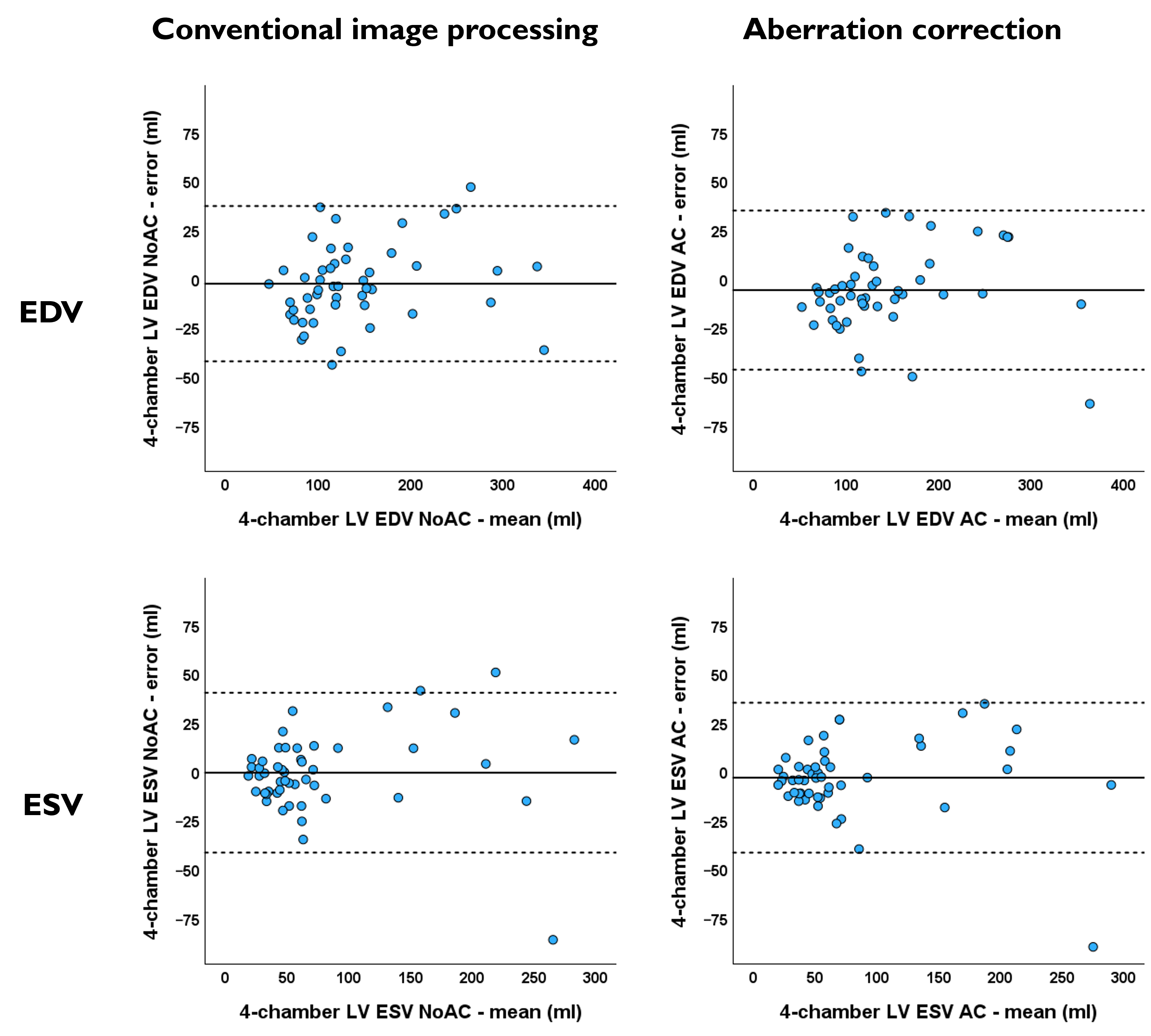

Supplement: qyae140_Supplementary_Data [file qyae140_Supplementary_Data.zip › Supplementary figure 3.tif]

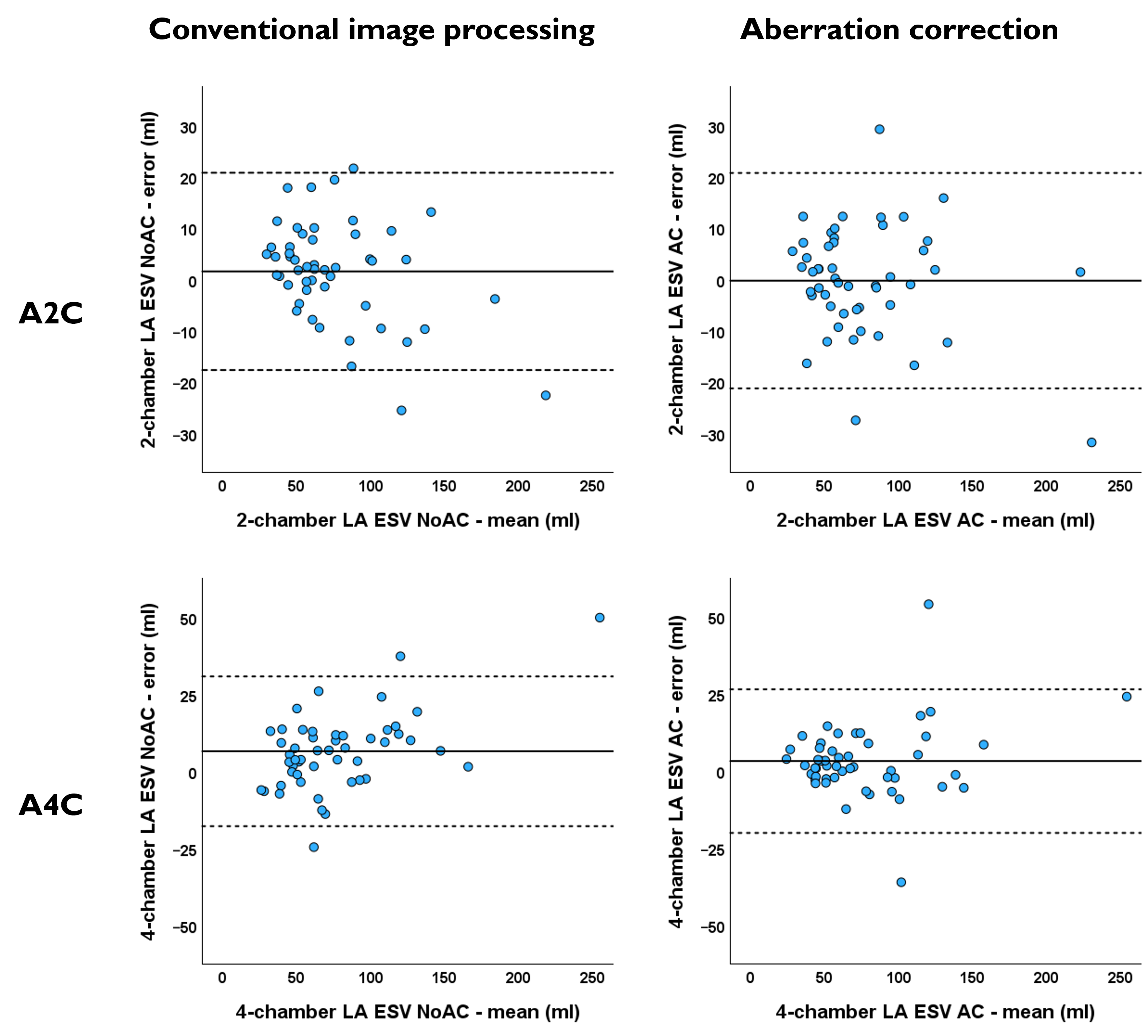

Supplement: qyae140_Supplementary_Data [file qyae140_Supplementary_Data.zip › Supplementary figure 4.tif]

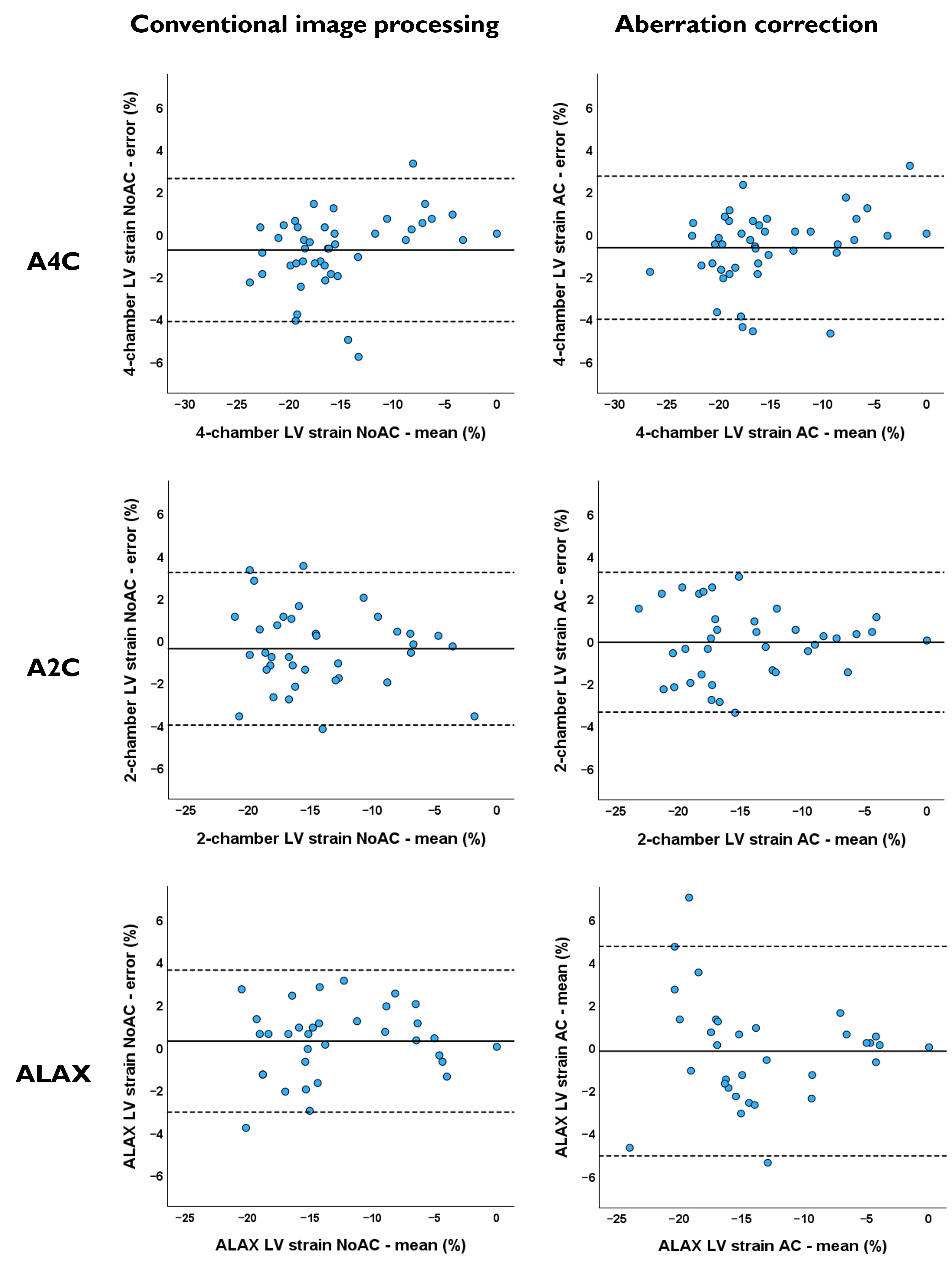

Supplement: qyae140_Supplementary_Data [file qyae140_Supplementary_Data.zip › Supplementary figure 5.tif]
